# Aquatic Acoustic Metrics Interface Utility for Underwater Sound Monitoring and Analysis

**DOI:** 10.3390/s120607438

**Published:** 2012-05-31

**Authors:** Huiying Ren, Michele B. Halvorsen, Zhiqun Daniel Deng, Thomas J. Carlson

**Affiliations:** 1 Pacific Northwest National Laboratory, Hydrology Group, P.O. Box 999, Richland, WA 99352, USA; E-Mail: Huiying.ren@pnnl.gov; 2 Pacific Northwest National Laboratory, Marine Science Laboratory, 1529 West Sequim Bay Road, Sequim, WA 98382, USA; E-Mail: Thomas.carlson@pnnl.gov

**Keywords:** underwater sound monitoring, software, noise recording, audiograms, sound exposure level

## Abstract

Fishes and marine mammals may suffer a range of potential effects from exposure to intense underwater sound generated by anthropogenic activities such as pile driving, shipping, sonars, and underwater blasting. Several underwater sound recording (USR) devices have been built to acquire samples of the underwater sound generated by anthropogenic activities. Software becomes indispensable for processing and analyzing the audio files recorded by these USRs. In this paper, we provide a detailed description of a new software package, the Aquatic Acoustic Metrics Interface (AAMI), specifically designed for analysis of underwater sound recordings to provide data in metrics that facilitate evaluation of the potential impacts of the sound on aquatic animals. In addition to the basic functions, such as loading and editing audio files recorded by USRs and batch processing of sound files, the software utilizes recording system calibration data to compute important parameters in physical units. The software also facilitates comparison of the noise sound sample metrics with biological measures such as audiograms of the sensitivity of aquatic animals to the sound, integrating various components into a single analytical frame. The features of the AAMI software are discussed, and several case studies are presented to illustrate its functionality.

## 1. Introduction

Both abiotic and biotic sounds are extremely important to fishes and other marine mammals for almost all life activities. Survival of not only individual animals but of whole populations can be affected by sound [1–6]. The level of abiotic sound from anthropogenic sources has increased significantly over the past few decades. Research conducted to assess the impacts of sound generated by pile driving, seismic surveys, and other maritime construction and military activities has shown that aquatic animals suffer a range of potential effects, including death, from exposure to these sounds [7–10]. Some effects, including hearing temporary threshold shifts or behavioral changes, are not directly lethal but may lower an animal's chance of survival by any number of mechanisms such as increased susceptibility to predation or reduced foraging success.

The physiological and behavioral impacts of sound on fishes and marine animals can be complicated, and substantial effort is required for their investigation. Investigation of the effects of sound requires measurement of the sound to determine its characteristics such as frequency content and loudness. Several underwater sound recording devices (USRs) have been built to record underwater sound [11–16]. The recorded sound samples must be processed to obtain measurements in physical units that describe the sound. Several audio editing and processing software programs such as Audacity, GoldWave, and WAVELAB have been developed to perform various sound signal processing tasks. We determined that these programs did not meet our needs because they did not integrate the calibration information for the sound acquisition system, computation of biologically important sound metrics, and aquatic animal audiograms into a single analytical frame. Specifically, available software lacked the ability to convert voltage (units of recorded data) into physical units such as Pascals, did not provide functions to calculate sound metrics such as sound pressure level (SPL) and sound exposure level (SEL), and did not permit direct comparison of computed metrics with animal audiograms. In addition, most available programs were unable to batch process sound sample files, which is very useful when dealing with large numbers of sound files.

The purpose of this study was to build a program controlled by an easy-to-use graphical user interface (GUI) that could be used to efficiently post-process underwater sound files recorded by the USRs and other devices. In addition to the features mentioned so far, the Aquatic Acoustic Metrics Interface (AAMI) was designed to implement some of the same sound file editing functions found in other commercial software, as well as offer unique features unavailable in other programs.

The main reason underwater sound is measured is to obtain data that can be compared with information that describes biologically important functions, such as, the ability of an animal to hear a sound or to compare the characteristics of the observed sound with physiological or behavioral impact thresholds of exposed animals. For example, an audiogram is a standard way of representing an animal's hearing sensitivity over a frequency range. AAMI makes it easy to directly compare the characteristics of observed sound with the hearing sensitivity of animals of interest by plotting the spectra of the sound sample and the species' audiogram together in a single figure.

## 2. Program Description

The AAMI program was written in MATLAB (Version 7.11, Release 2010b, The MathWorks, Natick, MA, USA) under the Windows operating system (Microsoft Corporation, Redmond, WA, USA). At this time, it has been tested on 32-bit and 64-bit Windows operating systems. To obtain the best user interface presentation, the computer monitor resolution should be at least 1,280 × 1,024 pixels. The MATLAB signal processing toolbox is a component inserted into AAMI to filter and calculate spectrograms and power spectral densities (PSDs) for sound samples. A significant benefit of AAMI is the open source code format, which can be readily modified to meet additional processing, computation, and display needs as they are identified.

**Figure 1.**
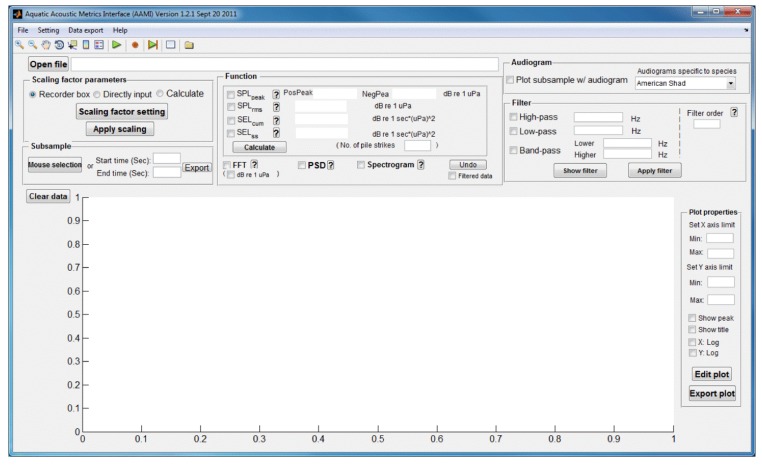
Main window of AAMI.

The operation and primary functions of AAMI are described in this section. Once AAMI is launched, the main window of AAMI pops up (Figure 1). The main menu and shortcut toolbars are located below the title bar. Below the main menu toolbar, the interface displays several component blocks including sound file upload, signal graphing, sound metric value calculation, unit conversion, filter application, audiogram selection, save settings option, interactive plotting, and data export of results and graphs. AAMI can process two channels of sound data simultaneously. If a sound file has more than two channels of data, the data for the first two channels in the file are chosen for processing and any other channels of data are ignored.

### 2.1. Upload of Sound File and Settings

AAMI can process sound files in .wav, .aif, and .mp3 formats. In the main window, the user can select a sound file for upload. The data source path will be shown in the uppermost white box on the left-hand side of the screen, and the signal is plotted in the time domain with voltage on the *y*-axis and time in seconds on the *x*-axis. The upper limit for file size is 2 gigabytes (GB); AAMI automatically checks file size and alerts the user if file size exceeds this limit. Depending on the amount of memory in the users' computer, AAMI could become sluggish and may freeze when loading a file larger than 1 GB. In addition, for sequential processing of files, AAMI will automatically load a settings file, which applies default values for some parameters on the scaling factor panel. For each file processed, the user can change the default settings, load another settings file, or save the current settings file under a new name.

### 2.2. Physical Unit Conversion

For a signal recorded by USRs and other devices, the typical output unit for the original data file is voltage. AAMI, in the *Scaling factor parameters* section of the user interface, provides three options for calculating the scaling factor to convert voltage to physical units:

1. The *recorder box* setting is specifically for sound files recorded from a USR [13] designed by Pacific Northwest National Laboratory (PNNL). A few advantages of PNNL's USR include (a) two input channels to record signals simultaneously; (b) flexibility of alternating between hydrophones or other sensors for different purposes; (c) a sampling frequency of 96 kHz, providing a wide dynamic range; (d) portability, low cost, and ease of implementation. The USR recording parameters used when collecting sound files such as the recorder serial number, channel number, gain, and hydrophone sensitivity (in V/Pa or dB re 1V/Pa) can be entered using the interface. With the provided parameters, AAMI will automatically calculate the scaling factor needed to transform electrical units to physical units.
2. The *Directly input scaling factor* setting provides a simple way to apply a scaling factor for sound files from any USR. The input scaling factor unit is Pa/V.
3. The *Calculate scaling factor setting* can be used for audio files obtained from any USR. Two parameters need to be specified: the hydrophone sensitivity in dB or V/Pa units and the receiver gain, entered in dB or x (times) units.

**Figure 2.**
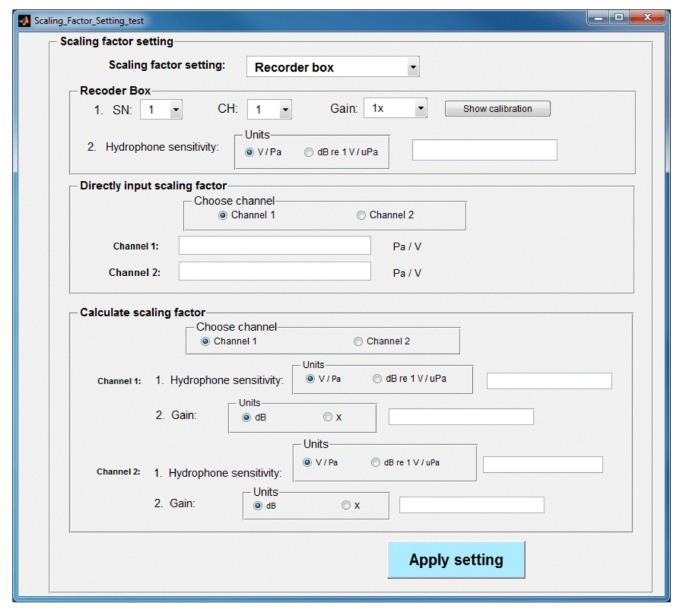
Scaling factor setting window of AAMI.

After the user selects one of the above three options, a new window will pop up for entry of the necessary values while the other two methods are disabled (Figure 2).

### 2.3. Subsample Selection

Another important feature of the AAMI program is selection of a subsample of data from a long signal. A data subsample may be selected in one of two ways: by using the computer mouse to select a portion of the signal shown in the interface plot area or by entering a time range in the editing boxes within the *Subsample* component portion of the interface menu.

### 2.4. Calculation Function

For a subsample or the complete sound file, AAMI can calculate the following seven parameters:

1. Peak sound pressure level (*SPL_peak_*) is the maximum absolute amplitude value in the signal during a specified time interval:
  (1) $${\text{SPL}}_{\text{peak}}=20\log_{10}\left(\frac{P_{\text{peak}}}{1\cdot \mu Pa}\right)$$
  where *P_peak_* is the peak pressure and units are dB re 1 µPa.
2. Root mean square (RMS) sound pressure level (*SPL_rms_*) is the log transformed square root of the average square pressure of the signal over a specific time interva:
  (2) $${\text{SPL}}_{\text{rms}}=20\log_{10}\left(\frac{P_{\text{rms}}}{1\cdot \mu Pa}\right)$$
  where *P_rms_* is the RMS pressure and units are dB re 1 µPa.
3. Sound exposure level (*SEL*), also referred to as single-strike SEL (*SEL_ss_*), is the squared sound pressure integrated over the duration of a signal:
  (3) $${\text{SEL}}_{ss}=10\log_{10}\left(\frac{\sum_{i=1}^{n}P_{i}^{2}(t)}{P_{\text{ref}}}\cdot \Delta t\right)$$
  where *P* is pressure, *P_ref_* is reference pressure, Δ*t* is the inverse of sampling frequency, and units are dB re 1µPa^2^·s.
4. Cumulative sound exposure level (*SEL_cum_*) is the summation of multiple impulsive or transient signals. SEL is calculated by summing the cumulative pressure squared over the time of the event and normalized to one second. This metrics accounts for both negative and positive pressures. Although not usually the case, when the *SEL_ss_* is the same for all impulses *SEL_cum_* can be calculated as shown in Equation (4):
  (4) $${\text{SEL}}_{\text{cum}}={\text{SEL}}_{ss}+10\log_{10}\left(\text{No. of impulsive sounds}\right)$$
  where units are dB re 1 µPa^2^·s.
5. Fast Fourier transform (FFT) decomposes a signal into its frequency components. AAMI implements the FFT and the inverse transform pair by:
  (5) $$X(k)=\sum_{j=1}^{N}x(j)\omega_{N}^{\left(j-1)(k-1\right)}$$
  (6) $$x(j)=\left(\frac{1}{N}\right)\sum_{k=1}^{N}X(k)\omega_{N}^{-(j-1)\left(k-1\right)}$$
  where *N* is the signal length and ω_N_ = e^(2πi)/N^ is the *N^th^* root of unity.
6. Power spectral density (PSD) is the power in the signal per unit frequency over the duration of the signal. AAMI computes the PSD using Welch's method [17], which is based on Bartlett's method and divides the signal into several overlapping segments that are windowed. For this interface, the defined window function is a hamming window [18], which is optimized to decrease the amplitude of side lobes in the spectrum. Users can define the length of the FFT and the overlap for the estimation of the PSD, which is normally set to 50%.
7. Spectrogram is an image showing the level of energy (spectral density) across frequencies over time. AAMI uses the short-time Fourier transform (STFT) [18,19] to calculate the spectrogram because most recorded sound files are sequential. AAMI provides a computation control window that allows users to enter the length of the FFT and the window function overlap to compute the time-frequency spectrogram.

### 2.5. Audiogram

**Figure 3.**
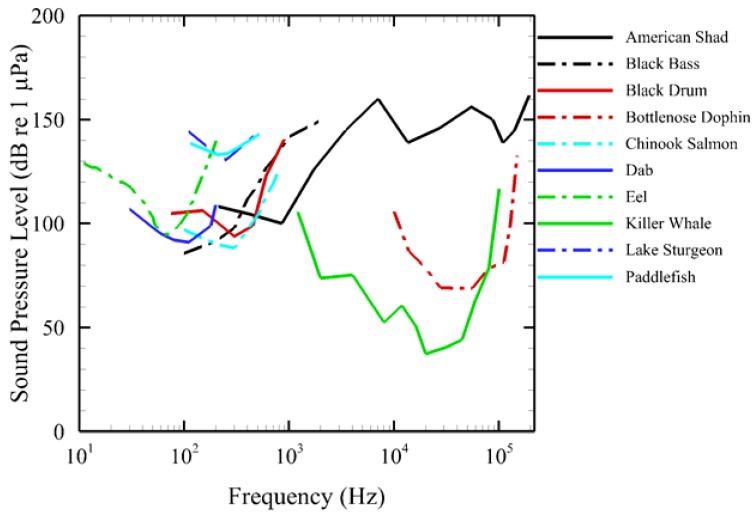
Audiograms of the 10 species of fish and marine mammals in the AAMI audiogram library [20–28].

An audiogram is a standard way of presenting an animal's hearing sensitivity in functional form as a variable threshold over a frequency range. Currently, AAMI contains a library of audiograms (Figure 3) for a number of fish and marine mammal species: American shad [20], black bass [21], black drum [22], bottlenose dolphin [23], Chinook salmon [24], eel [25], dab [26], lake sturgeon and paddlefish [27], killer whale [28]. We selected these audiograms from existing literature and did not evaluate their quality independently. Additional audiograms may be entered when they become available.

### 2.6. Filter

Another important feature contained in AAMI is the ability to filter sound files before processing them. AAMI permits an audiogram to be used as a filter in addition to a selection of other, more common filters given below:

1. high-pass filter, which removes frequencies lower than a specified value;
2. low-pass filter, which removes frequencies greater than a specified value;
3. bandpass filter, which removes frequencies outside a given range of values.

### 2.7. Plot Edit and Export

Users can edit a sound signal or other plots by adjusting the range and scaling for *x*- and *y*-axes, and can turn on/off the appearance of text indicating peak values and plot title. Moreover, AAMI can export the working graphic into different types of output files such as .tif, .eps, and .jpg.

### 2.8. Batch Process

AAMI can sequentially batch-process an unlimited number of .wav files using a setting file. As mentioned before, for large files, AAMI might freeze during loading and processing the data. To avoid this situation, break long duration signals into a series of shorter duration files that are less than 1 GB in size and use the AAMI batch-processing capability to process the signal. The batch-processing function loads the .wav files and outputs user-defined plots or data.

## 3. Example Application

There is concern for the potential effect of noise generated by tidal turbines on nearby organisms of ecological importance. A laboratory exposure-response study was conducted to investigate the response of juvenile Chinook salmon (*Oncorhynchus tshawytscha*) to tidal turbine noise. This species and age of salmon were selected for test because they are an ESA listed species and because they travel through Admiralty Inlet in Puget Sound as both juveniles and adults and could be exposed to tidal turbine and other noise [29].

Sounds that are intense and/or of long duration have been shown to affect the auditory system of fish [24,30,31]. If the auditory system is affected by sound, it is often expressed as a temporary shift in hearing threshold [24,32,33]. Many types of anthropogenic sources, such as shipping and tidal turbines, produce low-frequency sounds that fall into the auditory detection range of fish. Marine and hydrokinetic turbines generate noise energy that falls within the most sensitive portion of the frequency range of most fishes' auditory systems. Any of these sources could cause damage to auditory sensitivity. A temporary loss of sensory function, like hearing, could have implications for survival because the fish may be at a decreased capacity to detect predators, prey, and/or conspecifics. Furthermore, many underwater sounds can cause tissue damage such as hematomas, internal hemorrhaging, and ruptured swim bladders.

**Figure 4.**
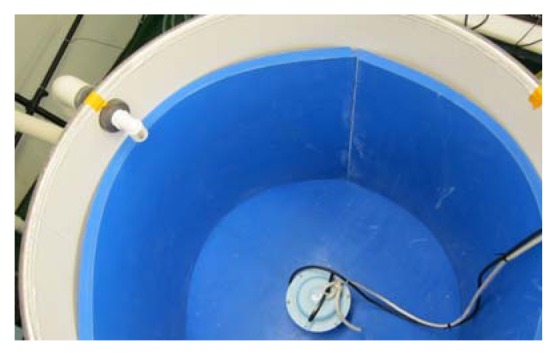
The noise exposure test tank.

In our response tests, salmon were exposed to simulated tidal turbine noise continuously for 24 h around a SPL_rms_ of 159 dB re 1 µPa to measure the effects on their hearing and tissues. That level corresponded to the source level of a prototype turbine, estimated from measurements of an operating turbine [34]. After exposure to the noise, fish were assessed at four different times for tissue damage and for changes in hearing sensitivity. The noise exposure test tank was a 500-L round aluminum tank lined with a blue anechoic material (Aptflex F48, Precision Acoustics Ltd., Dorchester, UK) to stiffen the walls and create a uniform sound field (Figure 4). A UW30 speaker (Lubell Labs Inc., Columbus, OH, USA) was placed in the center of the tank's bottom, connected to a Sony PCM-D50 recorder that played back simulated tidal turbine noise. A hydrophone (Model TC4013, Reson, Inc. Goleta, CA, USA; sensitivity −211 dB re 1 V/µPa) was positioned in the center of the water in the tank at a depth of approximately 30 cm. This placement of the hydrophone permitted measurement of the sound to which test fish were exposed. The hydrophone was calibrated by the manufacturers for the frequency range of 250 Hz to its usable frequency ranges. It was also recalibrated in PNN's in-house A2LA accredited underwater acoustic facility [35].

**Figure 5.**
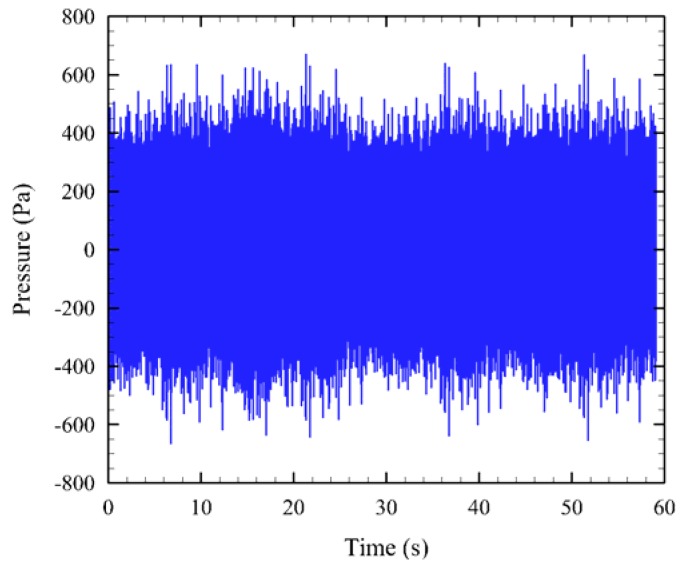
An example of a 1-minute segment of simulated tidal turbine sound in the time domain.

**Figure 6.**
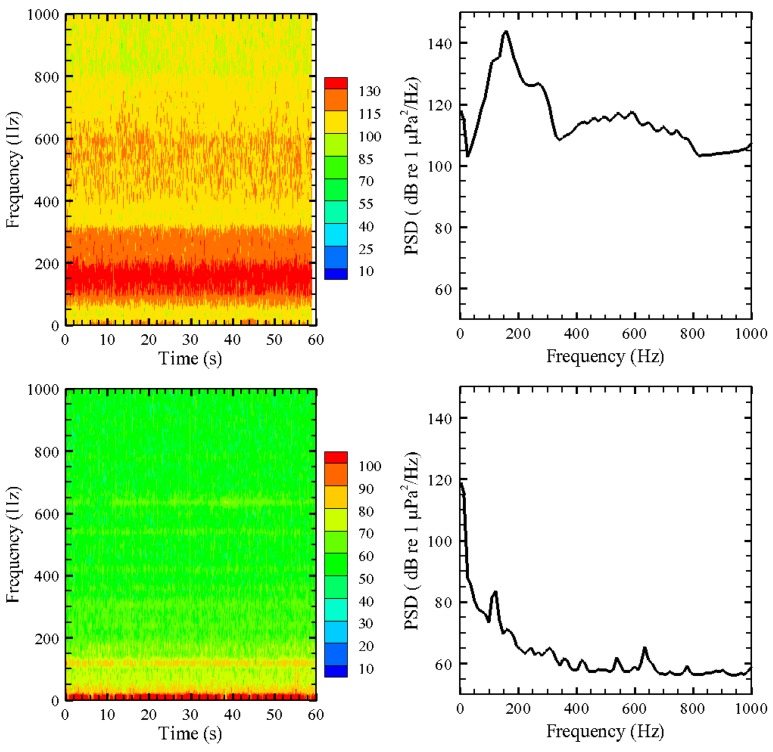
Spectrogram and power spectral density for exposure signal and control. Upper left: treatment 4, 1-minute spectrogram. Upper right: treatment 4, power spectral density. Bottom left: treatment control, 1-minute spectrogram. Bottom right: treatment control, power spectral density.

AAMI was used to post-process all exposure and control (*i.e.*, ambient) sound signals. The data for six 24-h tests were processed. The sound exposure signal, which was on continuously through the 24-h exposure period, was sampled using a USR in 1-min segments to keep file sizes manageable. All the 1-min sound files (exemplified in Figure 5) were batch-processed to estimate their *SPL_peak_* and *SPL_rms_* values. For each day, a random 1-min segment was further processed to obtain spectrogram and power spectral density (PSD) plots (Figure 6).

The Pacific Northwest National Laboratory animal facilities used in this research are Association for Assessment and Accreditation of Laboratory Animal Care (AAALAC)-certified; fish were handled in accordance with federal guidelines for the care and use of laboratory animals. The Institutional Animal Care and Use Committee at Battelle-Pacific Northwest Division specifically approved protocols for this study.

**Figure 7.**
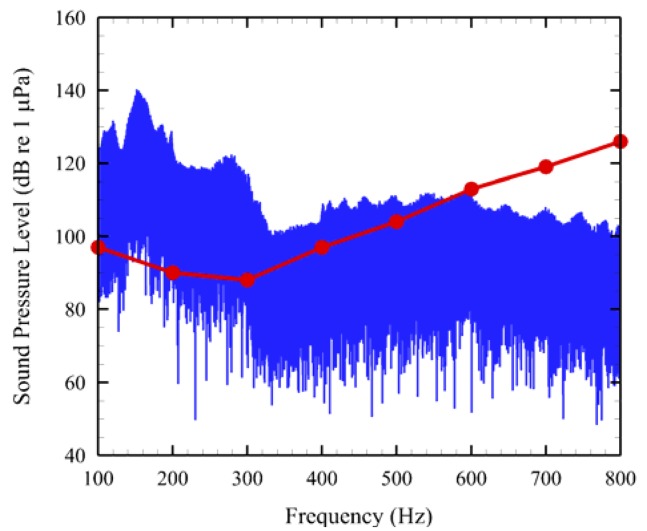
AEP derived audiogram for juvenile Chinook salmon overlaid on the sonogram of a noise sample.

One of AAMI's unique features was also applied to a 1-min sound sample (Figure 7). An audiogram derived from the evoked auditory potential (AEP) of a juvenile Chinook salmon [24] was overlaid on the noise sample. The red trace is the audiogram. Regions in which the red line is below or covered by the blue noise signal clearly show which auditory frequencies that would most likely be affected by tidal turbine noise (e.g., 100–500 Hz).

## 4. Conclusions

AAMI has been verified, with internal and external testing, to provide functionality, processing, and computation functionalities that are not available in other audio editing software programs. The AAMI GUI facilitates importing and processing sound signals in various sound file formats. AAMI was constructed to perform common sound file editing functions as well as to provide some unique features customized for use in scientific sound processing. AAMI has the capabilities to read two-channel sound signal recordings, convert the input data from engineering units to physical units (such as volts to pascals) apply user entered calibration information, batch-process sound files, and calculate important acoustic metrics such *SPL_peak_*, *SPL_rms_*, *SEL_ss_*, and *SEL_cum_* The output metrics of AAMI can help drive the standardization of sound analysis with regard to anthropogenic sound sources and their effects on marine life. All of AAMI's design capabilities, functions, and applications make AAMI a powerful tool that is relatively straightforward to employ. AAMI brings together many functions into one source that otherwise required the user to be an expert programmer to generate the metrics, graphs, comparisons, and output elements that AAMI can produce.

Furthermore, the pairing of the PNNL-USR [13] with the software utilities of AAMI provides a powerful combination of tools. This combination creates a manageable tool set that is easy to set up, deploy, collect sound data, and analyze the sound data without programming skills or the need of other programs. This tool set was designed with the intention of providing capabilities with which to standardize sound recordings along with the processing and analysis of acoustic signals and the reportable sound metrics. The combination of these tools can help to make the process of assessing anthropogenic sounds and their effects much more manageable.
